# Genome size distributions in bacteria and archaea are strongly linked to evolutionary history at broad phylogenetic scales

**DOI:** 10.1371/journal.pgen.1010220

**Published:** 2022-05-23

**Authors:** Carolina A. Martinez-Gutierrez, Frank O. Aylward

**Affiliations:** 1 Department of Biological Sciences, Virginia Tech, Blacksburg, Virginia, United States of America; 2 Center for Emerging, Zoonotic, and Arthropod-borne Pathogens, Virginia Tech, Blacksburg, Virginia, United States of America; Universidad de Sevilla, SPAIN

## Abstract

The evolutionary forces that determine genome size in bacteria and archaea have been the subject of intense debate over the last few decades. Although the preferential loss of genes observed in prokaryotes is explained through the deletional bias, factors promoting and preventing the fixation of such gene losses often remain unclear. Importantly, statistical analyses on this topic typically do not consider the potential bias introduced by the shared ancestry of many lineages, which is critical when using species as data points because of the potential dependence on residuals. In this study, we investigated the genome size distributions across a broad diversity of bacteria and archaea to evaluate if this trait is phylogenetically conserved at broad phylogenetic scales. After model fit, Pagel’s lambda indicated a strong phylogenetic signal in genome size data, suggesting that the diversification of this trait is influenced by shared evolutionary histories. We used a phylogenetic generalized least-squares analysis (PGLS) to test whether phylogeny influences the predictability of genome size from dN/dS ratios and 16S copy number, two variables that have been previously linked to genome size. These results confirm that failure to account for evolutionary history can lead to biased interpretations of genome size predictors. Overall, our results indicate that although bacteria and archaea can rapidly gain and lose genetic material through gene transfers and deletions, respectively, phylogenetic signal for genome size distributions can still be recovered at broad phylogenetic scales that should be taken into account when inferring the drivers of genome size evolution.

## Author summary

The evolutionary forces driving genome size in bacteria and archaea have been subject to debate during the last decades. Typically, independent comparative analyses have suggested that unique variables, such as the strength of selection, environmental complexity, and mutation rate, are the main drivers of this trait, without considering for potential biases derived from shared ancestry. Here, we applied a phylogeny-based statistical approach to assess how tightly genome size in bacteria and archaea is linked to evolutionary history. Moreover, we also evaluated the predictability of genome size from the strength of purifying selection and ecological strategy on a broad diversity of bacteria and archaea genomes under a phylogenetic comparative framework. Our approach indicates that despite the ability of bacteria and archaea to rapidly exchange genes, a strong phylogenetic signal to genome size distributions can be recovered at broad phylogenetic scales.

## Introduction

Bacterial and archaeal genomes are densely packed with genes and contain relatively little non-coding DNA, and therefore an increase in genome size is directly translated into more genes [1–3]. In contrast, multicellular eukaryotes generally show genome expansion due to the proliferation of noncoding-DNA as a consequence of high genetic drift [2]. The Depletion of non-functional elements in prokaryotes is explained through the bias towards more deletions than insertions; newly acquired or existing genes are removed if selection on those genes is insufficient for their maintenance in the population [4–6]. Although narrowly constrained when compared with eukaryotes, prokaryotic genome sizes still vary by over one order of magnitude. Assuming an intrinsic deletion bias across all prokaryotes, it remains unclear what evolutionary forces determine which genes are maintained and which are lost, and what determines the variability of genome sizes across the broad diversity of bacteria and archaea.

Multiple individual factors have been hypothesized to be primary drivers of genome size in bacteria and archaea. Early studies suggested that effective population size (*Ne*) may be the primary force that determines genome size and fluidity in prokaryotes [7,8]. For example, genome reduction has been observed in host-dependent bacteria that have small *Ne* and correspondingly high levels of genetic drift due to population contractions. Under such evolutionary constraints, slightly deleterious deletions accumulate and cause overall genome reduction [9–13]. Paradoxically, later studies focusing on abundant free-living planktonic lineages in the ocean suggested that genome reduction can also be observed in bacteria with larger *Ne* that experience strong purifying selection [14–17]. In this case selection favors genomic economization, such as the removal of paralogs and intergenic sequences. Factors other than *Ne* and the strength of purifying selection have also been postulated to play a role in determining prokaryotic genome size. Recently, one study suggested that environmental stress leads to genome streamlining in soil bacteria [18], and other genomics studies have suggested that habitat complexity and ecological strategy [19], as well as the capability to use oxygen [20] may also play major roles in determining genome size in bacteria and archaea [19]. Mutation rate has also been proposed to be a major factor determining genome size [21,22]. In particular, it was suggested that a high mutation rate would be the primary cause of genome reduction in both streamlined and host-dependent bacteria due to the erosion of genes, loss of function, and subsequent deletion [21–23]. However, other studies analyzing the mutation rate of the abundant picocyanobacteria *Prochlorococcus* show estimates similar to *Escherichia coli*, casting doubt on the view that high mutation rates drive genome reduction in all cases [24,25]. Given the large number of forces that have been proposed to be primary determinants of genome size, it remains largely unknown whether genome size in prokaryotes is driven by unique variables, their interaction, or variables that have specific influence depending on the lineage.

Importantly, most statistical analyses exploring the association between genome size and other traits have typically not used phylogenetic comparative methods that are necessary when using species as data points. Shared evolutionary history may obscure the relationship between traits because the phylogenetic dependence between lineages leads to the violation of the statistical assumption of independence in residuals. Thus, conventional statistical methods can lead to overestimation of the strength of the association between traits [26,27]. In this study, we estimated the phylogenetic signal of genome size across a broad diversity of bacterial and archaeal genomes available on the Genome Taxonomy Database (GTDB) [28,29]. Although genome size has been shown to change rapidly in prokaryotes due to HGT and gene loss, we sought to test if this trait still bore a phylogenetic signal across broad phylogenetic scales. Moreover, because previous studies have suggested that effective population size or ecological niche are potential drivers of genome size [3,8], we evaluated whether correlations with these factors would change if evolutionary history was taken into account. Our work provides important insights into the complex mechanisms that shape genome size in bacteria and archaea, and the importance of considering shared evolutionary relationships when studying its evolution to avoid bias in the association between traits.

## Results and discussion

### Genome size distribution across major phyla of bacteria and archaea

**Fig 1:**
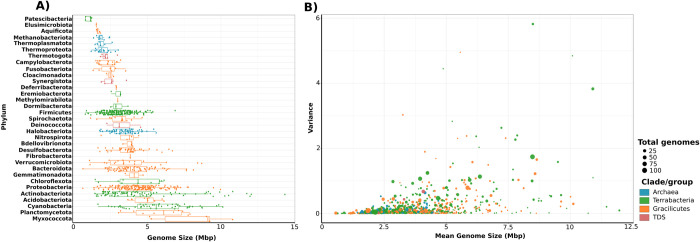
A) Distribution of genome size within bacteria and archaea taxonomic groups at the phylum level. First and third quantiles, as well as median are shown for each phylum distribution. B) Relationship between mean genome size and genome size variance for each genus cluster. Abbreviations: TDS = Thermotogota, Deinococcota, and Synergistota. Raw data for genome size can be found in S2 Data.

**Fig 2:**
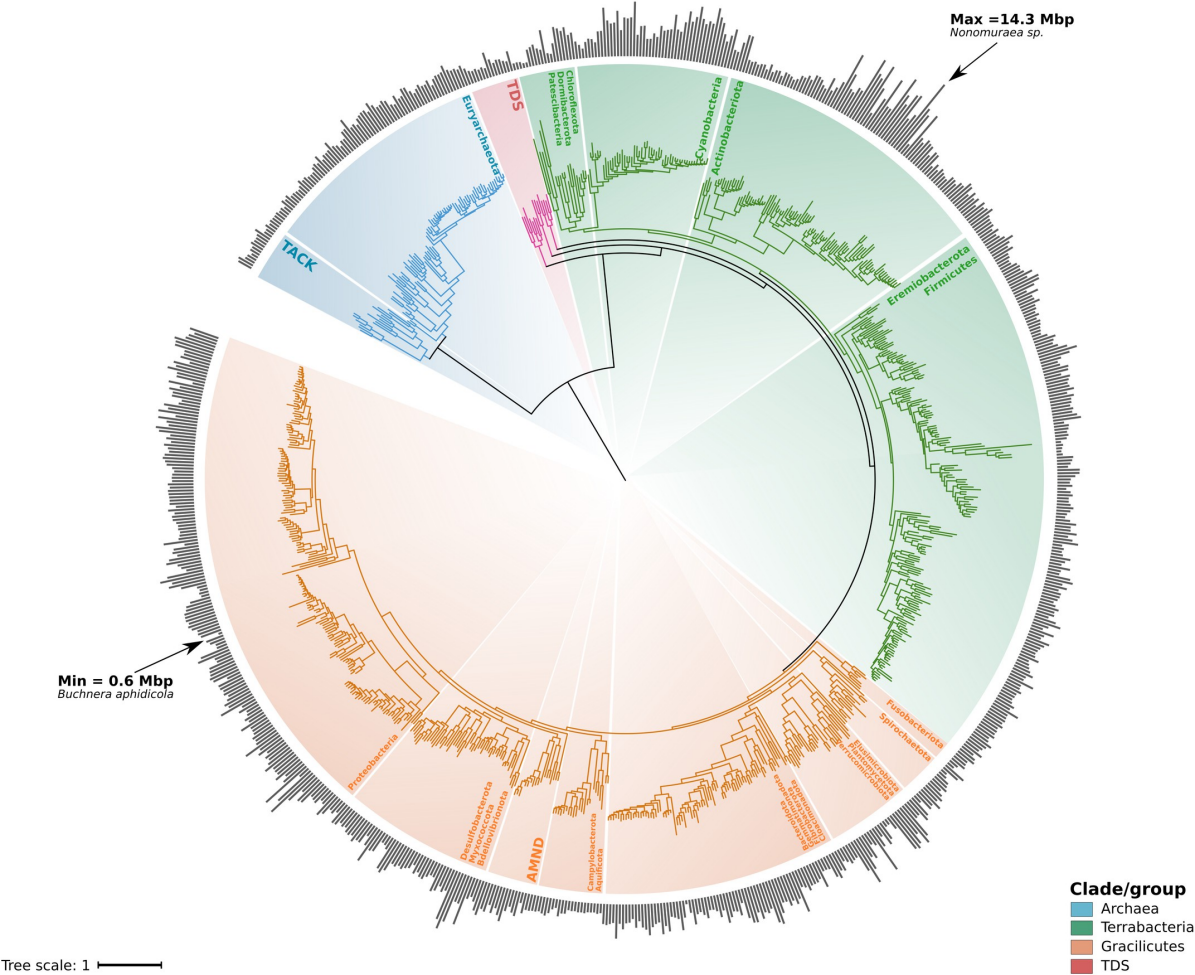
Genome size distribution across the Tree of Life of bacteria and archaea using one representative genome for each genus. Phylogenetic tree was built using a concatenated alignment of ribosomal and RNA polymerase sequences through a maximum likelihood approach and the substitution model LG+R10. Abbreviations: TACK = Thaumarchaeota, Aigarchaeota, Crenarchaeota and Korarchaeota; TDS = Thermotogota, Deinococcota, and Synergistota; AMND = Acidobacteriota, Methylomirabilota, Nitrospirota, Deferribacterota. Raw data for genome size can be found in S2 Data.

In order to explore the distribution of genome size across the Tree of Life of bacteria and archaea, and to measure phylogenetic signal across broad phylogenetic scales, we built a phylogenetic tree using one representative genome of 836 genera belonging to 33 phyla available on the GTDB. For the reconstruction of this phylogeny, we used a set of ribosomal proteins and RNA polymerase subunits that we have previously benchmarked [30]. The size of genomes in our analysis and across the phylogeny varied by almost two orders of magnitude (0.6–14.3 Mbp, Figs 1A and 2). The minimum and maximum corresponded to two bacterial lineages with contrasting lifestyles: the endosymbiont *Buchnera aphidicola* of the phylum Proteobacteria and the free-living Actinobacteria *Nonomuraea sp*. (Fig 1A and 2). The greatest within-phylum variation of genome size was observed for the phyla Actinobacteria and Cyanobacteria, whereas Patescibacteria had the shortest mean genome length (Fig 1A). We also evaluated the difference in genome size found within the genera used in our study, which we report here as the variance (Fig 1B) and the difference between the largest and smallest genomes within each genus (S1 Fig). Most of the genera used in our analysis (571 out of 863) showed a difference smaller than 1 Mbp, but some genera exhibited a wide range of genome sizes; for example the genera *Streptomyces* and *Nonomuraea* showed a difference of 6.29 and 6.06 Mpb between the smaller and the larger genomes, respectively (Figs 1B and S1). The large difference found between the largest and smallest genome of some of the genera in our dataset is consistent with previous observations of considerable differences in the genome size and genome content of many closely related taxa [31–34].

### Genome size in bacteria and archaea is strongly dependent on phylogenetic history at broad evolutionary scales

Although it is well known that genome size can vary markedly between closely-related bacteria and archaea [31–34], it is still possible that overall genome size distributions are linked to evolutionary history at broad phylogenetic scales, which we define here as anything broader than the genus level according the GTDB classification (Fig 2). Due to the shared evolutionary history of some lineages, traits of related groups often resemble each other more than when compared with randomly-selected species in the same phylogenetic tree (phylogenetic signal) [35–37]. We therefore sought to investigate the phylogenetic signal of genome size distributions in our genome dataset (Fig 2). Phylogenetic methods are needed to analyze these associations because any study involving statistical analyses and species as data points potentially violates the assumption of independence of residuals [26,38].

When studying phylogenetic signal, it is recommended to measure it at two different levels: 1) in traits’ raw data and 2) in the residuals resulting from statistical models (e.g., regressions) [39]. As a first approximation, we assessed whether genome size distribution data show phylogenetic signal by estimating Blomberg’s K [35] on the genome size of the GTDB genome dataset (Fig 2). Values of Blomberg’s K between 0 and 1 indicate that the sizes of closely related genomes resemble each other but less than expected under the Brownian Motion model (BM) of trait evolution, where trait variation is proportional to phylogenetic distance [26]. Conversely, a K of 1 is evidence of genome size variation according to the Brownian Motion expectation [35]. We observed phylogenetic signal in genome size data that is strong but different to what would be expected under the Brownian Motion model (BM) (K = 0.51, P = 0.001), suggesting that although genome size shows phylogenetic signal, variation is not fully explained through phylogenetic distance in our tree [40].

In addition, we tested the fit of different models of trait evolution for genome size, including Brownian Motion [40], Ornstein-Uhlenbeck [41], Early-Burst [42], a diffusion model, Pagel’s model [43], a drift model, and a white-noise model (non-phylogenetic signal) (Table 1). According to a likelihood ratio test performed (P<0.001 when compared with the next-best likelihood), Pagel’s model showed the best fit (Table 1) with a lambda value of 0.90 (P<0.001). The Pagel’s lambda (λ) represents how strongly phylogenetic relationships predict the observed pattern of variation of a trait at the tips of a phylogeny, and varies from 0 (no phylogenetic signal) to 1 (phylogenetic signal under BM) [43]. Although we obtained different estimates for Blomberg’s K and Pagel’s λ, we considered that λ is more reliable because this metric is more robust than Blomberg’s K in situations of erroneous branch lengths [44]. Our λ estimate supports our conclusion that genome size data in bacteria and archaea show phylogenetic signal. These findings indicate that genome size in bacteria and archaea does not evolve independently of broad evolutionary relationships. To confirm that our phylogenetic signal estimates are not unduly influenced by the phylogenetic scale that we examined, we repeated our analyses using a larger set of genomes consisting of multiple representatives for each genus (S1 Data) and we observed a similar trend (S1 Table), suggesting that the phylogenetic signal trend observed in genome size data is not the result of a biased taxonomic representation. Moreover, for our genus-level tree we estimated kappa (k) and delta (δ) on genome size data, two parameters that describe the mode of evolution of a trait (punctuated vs gradual) and the rate change across the phylogeny (acceleration vs deceleration), respectively [45]. Our estimates (k = 0.24 and δ = 3) are consistent with a gradual and late diversification of genome size in bacteria and archaea, which might indicate lineage-specific adaptations [43,45].

**Table 1:** Summary of model fitting for genome size data. We highlighted the model that showed the highest likelihood and the lowest AIC.

Model	Loglik	Parameters	AIC
Brownian motion	-1463.3	Sigma = 12.3 Root state = 2.7	2930.7
Ornstein-Uhlenbeck	-1420.7	alpha = 2.7 Sigma = 17.5 Root state = 3.1	2847.4
Early-Burst	-1463.3	a = 0 Sigma = 12.3 Root state = 2.7	2932.7
**Pagel’s model** *	**-1415.6**	**Lambda = 0.9** **Sigma = 6.2** **Root state = 2.7**	**2837.2**
Trend diffusion	-1447.7	Slope = 100 Sigma = 0.1 Root state = 2.9	2901.3
Drift	-1463.3	Drift = -99.9 Sigma = 12.2 Root state = 102.7	2932.7
White-noise	-1695.6	Sigma = 3.4 Root state = 3.9	3395.2

*Significantly higher likelihood when compared with the rest of the models tested according to the chisq test (P<0.001)

Because phylogenetic signal estimates can be biased due to sample size [46], we measured phylogenetic signal within each phylum (Fig 1A). Our results indicate that most of the phyla with a small sample size (<25 genomes) showed remarkably large or small K and λ values (S3 Fig), consistent with previous findings that small sample sizes lead to biased estimates [38,46]. We did not observe a linear increase in λ values with the number of genomes tested, however, suggesting that the large lambda estimate found in our overall genome size data is not associated with our large sample size (S3 Fig).

### Non-phylogenetic regression overestimates the effect of dN/dS on genome size

**Fig 3:**
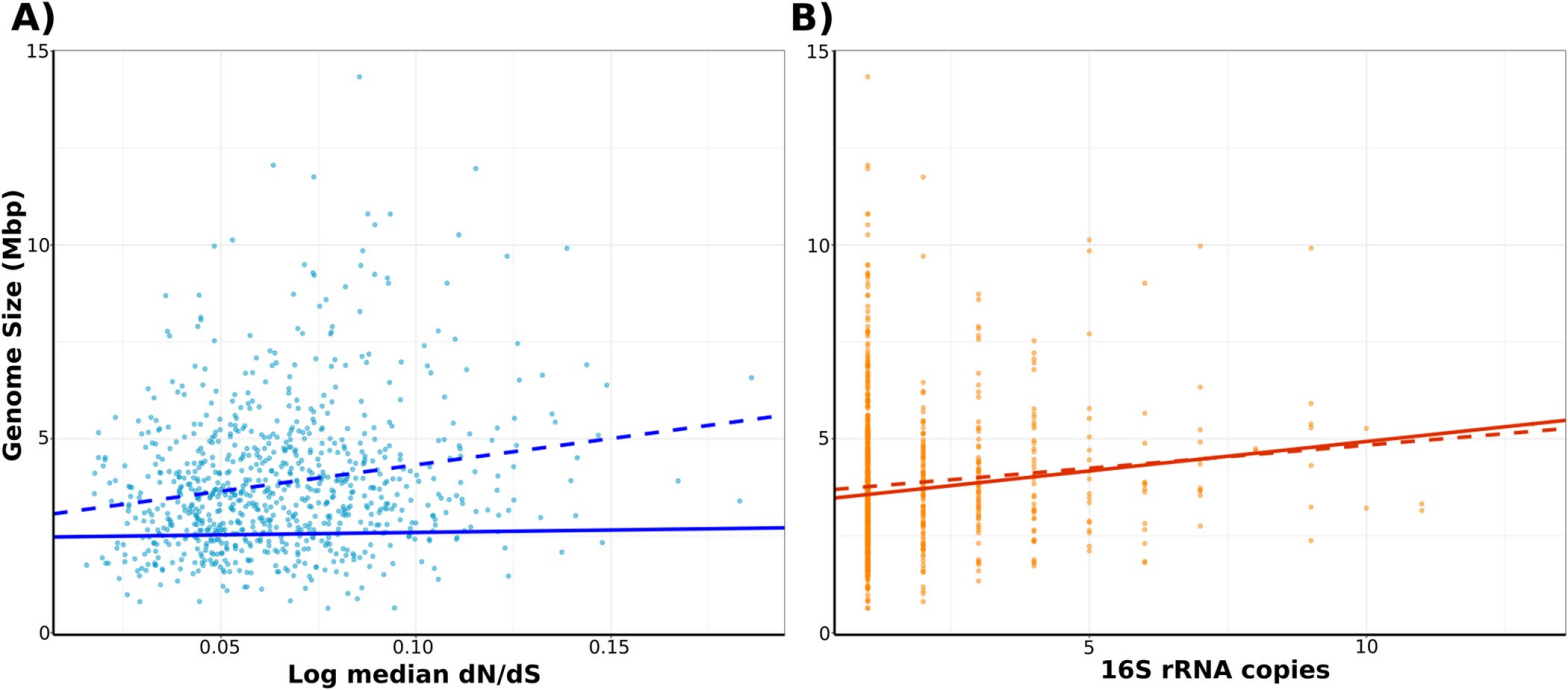
Relationship between genome size and genomic traits for bacteria and archaea using one representative genome for each genus. A) Regression line of the relationship between genome size and dN/dS ratio before (dashed line) and after (solid line) taking phylogenetic relationships into account through the Pagel’s model. B) Regression line of the relationship between genome size and 16S rRNA copies before (dashed line) and after (solid line) taking phylogenetic relationships into account through the Brownian Motion model. Parameters of the regression equation for both relationships can be found in Table 2. Raw data can be found in S2 Data.

We next explored whether the residuals resulting from the statistical association between genome size and other traits show phylogenetic signal. Previous studies have suggested that high levels of genetic drift are related with a decrease in genome size in bacteria [8,47]. However, such studies were based on a limited set of genomes available at the time and did not include a broad repertoire of streamlined genomes, which are notable for their small genomes and large effective population sizes [12,48]. We first investigated whether this trend is maintained when including a broader diversity of taxa by calculating pairwise dN/dS values for each genus in the GTDB genomes dataset. Our non-phylogenetic generalized least squares (GLS) showed a positive and significant but weak correlation between genome size and dN/dS (P<0.001, Pseudo-R^2^ = 0.04, Table 2, Fig 3A). This result contrasts with earlier studies reporting a strong relationship between genome content and dN/dS [8,47]; we attribute this large discrepancy to the broad taxonomic representation in our dataset, which includes small genomes under both strong purifying selection and genetic drift [12]. Interestingly, when considering phylogeny through the better-fitting Pagel’s model, our phylogenetic generalized least squares model (PGLS) showed poorer predictability and a non-significant relationship between both variables (P = 0.5, Pseudo-R^2^ = 0.0006, Table 2, Fig 3A). Similar results were found in a study that analyzed the phylogenetic signal associated with genome size across prokaryotes and eukaryotes [49,50]. In this previous study, authors showed that the phylogenetic signal found in genome size data caused a biased association between Ne.*μ* (approximated through nucleotide diversity) and other genetic traits, including genome size [49,50]. Our PGLS analysis indicates that not only does genome size data show phylogenetic signal, but that the residuals of our regression models also bear this signal (Table 2), confirming the need of assessment of phylogenetic-based methods when studying the evolution of genome size [46,51]. We also calculated the lambda parameter on our dN/dS data, and the value found (λ = 0.68; 95% CI = 0.56–0.77) indicates a relatively high phylogenetic signal for this variable, suggesting that phylogenetically related microorganisms tend to experience similar levels of selection. Altogether, these results suggest that correlations between dN/dS and genome size found previously are largely driven by poor sampling and artifacts that arise by not specifically accounting for the recent shared evolutionary history of many lineages [26].

**Table 2:** Statistics of the regression models relating genome size with dN/dS and 16S rRNA as predictor variables using Generalized Least Square and Phylogenetic Least Square analyses. We highlighted the models that were statistically significant (α = 0.05).

Model	Predictor variable	Kappa (95% CI)	Lambda (95% CI)	Delta (95% CI)	Slope	Intercept	P-val	AIC	R^2^
Generalized Least Square									
**Genome Size ~ Median dN/dS**	dN/dS	-	-	-	13.57	2.97	<0.001	3366.2	0.04*
**Genome Size ~ 16S rRNA copies**	16S rRNA copies	-	-	-	0.12	3.65	0.002	3387.7	0.01*
**Genome Size ~ Median dN/dS + 16S rRNA copies**	dN/dS + 16S rRNA copies	-	-	-	14.11/0.13	2.7	<0.001	3355.9	0.05*
Phylogenetic Generalized Least Square									
Genome Size ~ Median dN/dS	dN/dS	0.48 (0.39–0.58)	0.98^a^^,^^b^ (0.96–0.99)	2.44 (2.01–2.85)	1.26	2.46	0.5	2748.8	0.0006**
**Genome Size ~ 16S rRNA copies**	16S rRNA copies	0.49 (0.34–0.59)	0.98^a^ (0.96–0.99)	2.49 (2.06–2.9)	0.08	2.42	0.003	2740.268	0.01**
**Genome Size ~ Median dN/dS + 16S rRNA copies** ***	dN/dS + 16S rRNA copies	0.49 (0.40–0.59)	0.98^a,b^ (0.96–0. 99)	2.51 (2.08–2.93)	1.29/0.08	2.35	0.009	2741.79	0.01**

*Nagelkerke’s R^2^

** Multiple R square; percentage of variance explained between a null model and the actual model given that precise model of trait change

***Anova did not show significant differences between models Genome Size ~ 16S rRNA copies and Genome Size ~ Median dN/dS + 16S rRNA copies (P = 0.48)

^a^Significantly different from 0 (no phylogenetic signal)

^b^Significantly different from 1 (Brownian Motion expectation)

Although our results indicate that dN/dS is a poor predictor of genome size in bacteria and archaea (Fig 3A), it is worth mentioning that dN/dS only reflects recent evolutionary constraints due to saturation of substitutions at synonymous sites [52,53]. Therefore, we do not discount that genome reduction may be driven in part by processes such as population bottlenecks and periods of relaxed selection that happened in the past but are not reflected in dN/dS estimations. This scenario has been suggested for *Prochlorococcus*, in which the genome simplification observed in this clade could be the result of periods of relaxed selection experienced in the past [53].

### Ecological strategy plays a role in genome size evolution in bacteria and archaea

In addition to testing the effect of the strength of selection on genome size, we also assessed the predictability of genome size from 16S rRNA copies as an approximation to ecological strategy using both, GLS and PGLS. Previous studies have shown that copies of the *rrn* operon can be a predictor of the number of ribosomes that a cell can produce simultaneously, and that this reflects the ecological strategy in microorganisms [54,55]. A large number of *rrn* copies is associated with the ability to adapt quickly to fluctuating environmental conditions (i.e., “boom and bust” strategies) [56], while multiple *rrn* copies would confer a metabolic burden to slow-growing microorganisms living in stable or low-nutrients environments because of ribosome overproduction [54]. Similarly to what we observed for dN/dS, we found a weak, positive, and significant relationship between genome size and 16S rRNA copies when using GLS (P<0.001, Pseudo-R^2^ = 0.01, Table 2, Fig 3B). Interestingly, we still observed a significant relationship when accounting for the phylogenetic signal in the residuals through a PGLS analysis (P = 0.003, Pseudo-R^2^ = 0.01, Table 2, Fig 3B). However, the Pagel’s lambda of this model was not significantly different from 1 (Table 2), indicating that the residuals of this model show a distribution closer to the BM expectation. After fitting under the BM, we still observed a positive and significant relationship between genome size and 16S rRNA copies (P<0.001, Pseudo-R^2^ = 0.02). Although the predictability of 16S rRNA is weak under both BM and Pagel’s model, our findings suggest that environment complexity plays a role on genome size independently of phylogenetic relationships. This is consistent with the observation that larger genomes tend to inhabit environments with temporal variability and diversity of resources [57,58]. In addition to fitting our model using dN/dS and 16S rRNA copies individually as predictors, we fitted an additive model with both variables (Table 2). An ANOVA test showed that a model including both variables does not significantly improve the fit when compared with the model based on 16S rRNA copies as a unique predictor variable (P = 0.48).

### A hypothesis for the evolutionary processes that shape genome size in bacteria and archaea

According to our phylogenetic comparative framework (Tables 1 and 2 and Fig 3), lineages with recent shared evolutionary history tend to maintain similar sizes since the divergence from their common ancestor. Nevertheless the pattern of variation in genome size data (λ = 0.90) differs from what would be expected under the Brownian Motion model. This finding suggests that besides evolutionary relationships, there are other variables defining genome size in prokaryotes. Our results are consistent with the view that genome size in prokaryotes is the result of a complex interplay of multiple variables, including evolutionary history, past events such as population bottlenecks, and environmental complexity (substrates available, variability in environmental factors, biotic pressure, etc.), but it can still remain relatively stable at broad phylogenetic scales. Although several factors have been proposed to be singular drivers of genome size in prokaryotes, such as effective population size [59], ecological strategy [19,60], and mutation rate [21–23], our findings strongly suggest that genome size is a complex trait determined by the interaction of multiple variables, and that the relative importance of these factors may vary across lineages.

**Fig 4:**
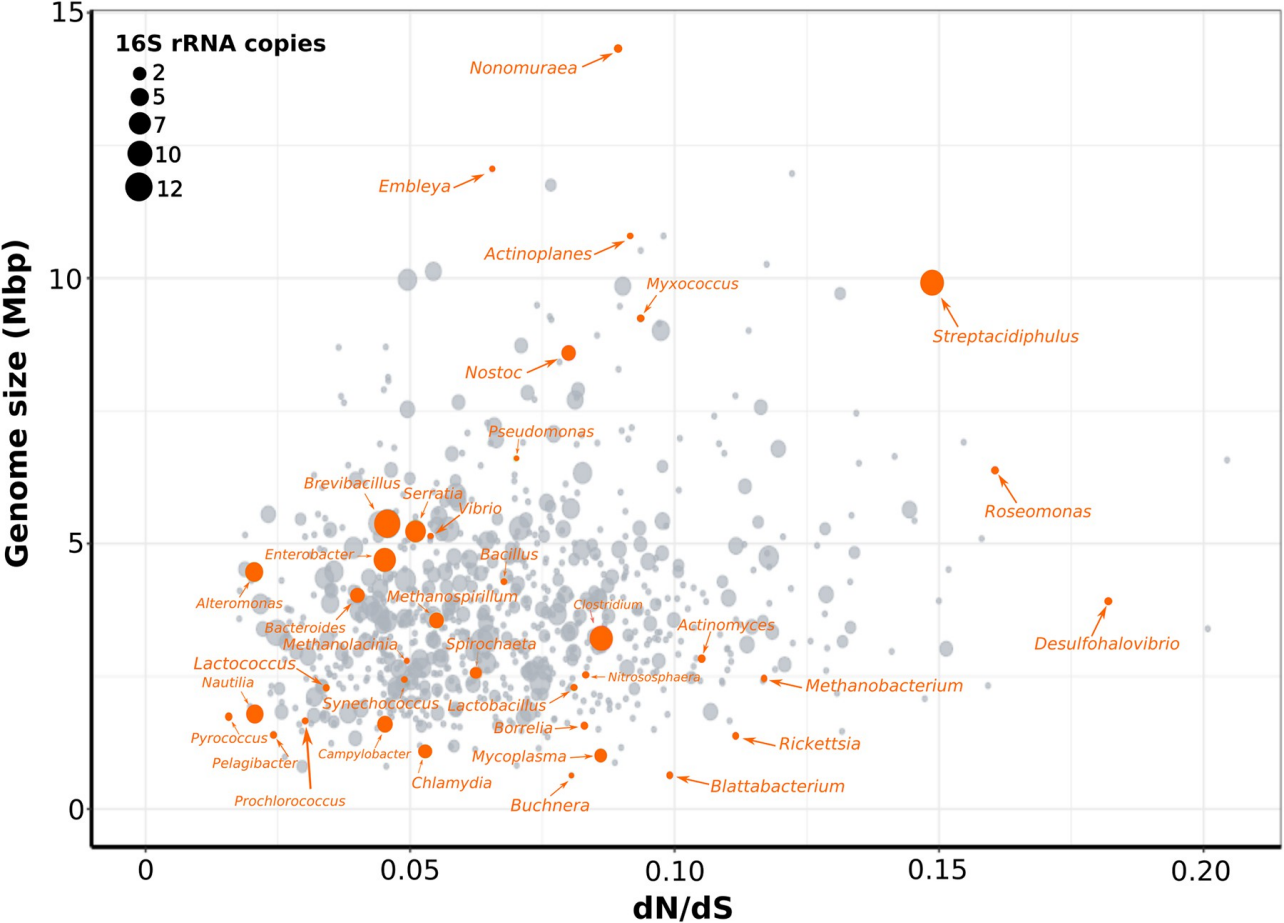
Relationship between genome size and dN/dS. dN/dS values represent the median estimate for each genus cluster. Dots represent a representative genome for each genus and size is equivalent to the number of 16S rRNA gene copies. Raw data can be found in S2 Data.

Phylogenetic signal estimates can vary across phylogenetic scales [61], and it is therefore possible that the strong phylogenetic signal found in our analyses is weaker or not observed at finer scales. This is particularly expected in clades that experience rapid genome turnover due to the acquisition and loss of genes through horizontal gene transfer events (HGT) and deletions, respectively. For example, genome contraction events are expected in endosymbionts like *Buchnera* and *Blattabacterium*, which are thought to derive from a large-genome ancestor [10], and are frequently undergoing bottlenecks and periods of diversity loss [9,10,62]. Such exacerbated loss of genes and diversity is enhanced by the nearly absent homologous recombination found in vertically transmitted endosymbionts [63]. These observations are consistent with the relatively high dN/dS value and small genome size that we observed for *Buchnera* and *Blattabacterium* (Fig 4). In contrast, some abundant marine clades inhabiting the open ocean such as *Prochloroccocus* and *Pelagibacter* have undergone long periods of adaptation and specialization to their stable environments [64,65]. The open ocean is characterized by chronically-oligotrophic nutrient conditions that are stable throughout the year [66], and genes that are under relaxed selection are therefore pseudogenized and lost [12]. The latter is supported by the unusual growth requirements and low number of transcriptional regulators found in *Pelagibacter*, which is expected to limit its response to changing environmental conditions [67,68]. Consistent with these observations, we observed low dN/dS values, small genome size, and fewer 16S rRNA for these streamlined bacteria (Fig 4). The small genomes observed in both endosymbionts and free-living planktonic lineages are therefore likely the result of distinct evolutionary processes, as previously proposed [17].

In contrast to the genome simplification observed in host-dependent and streamlined prokaryotes, genome expansion is expected in free-living lineages that inhabit complex environments like soils or sediments, where microenvironments with strikingly different abiotic conditions can be found millimeters apart [69]. Although temporal diversity declines and sweeps for specific gene variants are likely to occur in soil prokaryotes due to rapidly changing environmental conditions [69,70], larger genomes may be selected in these environmental realms due to variable abiotic and biotic constraints. Indeed, a study exploring the genes enriched in larger genomes of soil prokaryotes found a larger proportion of genes involved in regulation and secondary metabolism, and were depleted in genes related with translation, replication, cell division, and nucleotides metabolism when compared with smaller genomes [60]. These environmental and genomic findings are consistent with the large genome sizes, intermediate dN/dS, and multiple 16S rRNA copies estimated in our study for soil microorganisms of the genera *Actinomyces*, *Actinoplanes*, and *Myxococcus* (Fig 4), the latter showing complex fruiting body development [71]. It is interesting to note that the largest genomes analyzed in our study (>6 Mpb) tend to experience intermediate levels of purifying selection (dN/dS), suggesting that either extremely high or low purifying selection are not conducive to genomic expansion events.

## Conclusions

Despite the increase of genomes available on publicly available databases, the evolutionary processes and factors driving genome size and content in bacteria and archaea are continuously debated. Several studies have proposed ecological strategies, the strength of purifying selection, and mutation rate as prominent forces that individually determine prokaryotic genome size. Our statistical approach shows that, at broad phylogenetic scales, evolutionary history plays a large role in structuring genome size distributions across bacteria and archaea. Genome size is therefore not independent of phylogeny, and a failure to account for this can lead to misleading associations between traits. In some ways our finding of a strong phylogenetic signal to genome size in prokaryotes across broad evolutionary timescales is paradoxical given the well-known variability of prokaryotic genome size within species and between closely-related lineages [31–34]. These two realities need not conflict, however; for example it is possible that genome size fluctuates rapidly at short evolutionary timescales but remains relatively constant due to an overall balancing of gene gain and loss over long periods of time. The significant but poor relationship between genome size and 16S rRNA copies suggest that besides phylogenetic history, ecological strategy plays a role in shaping genome size in bacteria and archaea, although this single trait is insufficient to completely represent ecological strategies. Future studies will be necessary to evaluate the evolution of genome size on a lineage-by-lineage basis. However the strong phylogenetic signal observed in genome size data indicates that analyses involving this trait cannot consider species as phylogenetically independent, therefore phylogenetic relatedness should be assessed and taken into account in order to avoid simplified models and biased associations between traits.

## Material and methods

### Genomes compilation and phylogenetic reconstruction

In order to estimate the phylogenetic signal in genome size data at a broad phylogenetic scale, we compiled a genomes dataset that included a broad diversity of bacteria and archaea. All the representative genomes available on the Genome Taxonomy Database (GTDB) (Release 05-RS95; 17th July 2020) [28,29] were filtered based on completeness (> = 95%) and contamination (< = 5%) and then classified at the class levels. Genomes belonging to the phylum Patescibacteria (also known as Candidate Phyla Radiation or CPR) were filtered using the parameters completeness> = 80% and contamination< = 5%. After filtering and classification, classes with more than 500 genomes were randomly downsample to 500 genomes. The resulting genomes were clustered based on their taxonomic identity at the genus level and genera with fewer than two genomes were discarded from further analyses. Our final dataset consisted of 4380 genomes classified in 836 genera. For phylogenetic reconstruction, we randomly selected one genome from each genus (referred hereafter as GTDB genomes dataset) and used the MarkerFinder pipeline reported previously [30]. This pipeline consisted in the identification of 27 ribosomal proteins and three RNA polymerase genes (Ribosomal-RNAP set) [72] using HMMER3. The resulting individual sequences were aligned with ClustalOmega and concatenated. We trimmed the concatenated alignment with trimAl [73] using the option -gt 0.1. The Ribosomal-RNAP alignment was then used to build the phylogenetic tree with IQ-TREE 1.6.12 [74] with the substitutions model LG+R10 and the options -wbt, -bb 1000, and—runs 10 [75–77]. The resulting phylogeny was manually inspected on iTOL [78] (Fig 2). Raw phylogenetic tree is included in S1 Phylogeny.

### dN/dS estimation and *rrn* genes identification

To investigate whether the phylogenetic signal in genome size data leads to biased associations with other variables like the strength of selection and ecological strategy, we estimated the ratio of synonymous and nonsynonymous substitutions (dN/dS) within each genus cluster of our GTDB genomes dataset using two sets of conserved marker genes, checkm_bact and checkm_arch for bacteria and archaea, respectively [79]. Genomes used to calculate the dN/dS for each genus cluster are reported in S1 Data. The open reading frames (ORFs) retrieved from the GTDB were compared to the HMMs of the checkm_bact (120 marker genes) and checkm_arch marker (122 marker genes) sets using the hmmsearch tool available in HMMER v. 3.2.1 with the reported model-specific cutoffs [80]. We aligned the amino acid sequences for each marker gene and each genus cluster individually using ClustalOmega [81], and then converted amino acid alignments into codon alignments using PAL2NAL with the parameter—nogap [82]. We used the resulting codon alignments to estimate the pairwise ratio of synonymous and nonsynonymous substitutions for each pair of genomes using the maximum likelihood approximation (codeML) available on PAML 4.9h (runmode = -2) [83]. In order to avoid bias associated with divergence, dN/dS estimates with dS>1.5 were removed due to potential saturation. We also discarded pairwise comparisons with dS<0.1 because these might represent dN/dS values calculated from genomes of the same population. Moreover, dN/dS values >10 were considered artifactual [48]. Genomes with fewer than 25 dN/dS estimates remaining after filtering were discarded. We used the resulting median dN/dS of our representative genomes for further analysis. In order to examine the effect of genes’ selection on final dN/dS estimations, we randomly selected 40 genera and identified their core genes using CoreCruncher [84] using usearch [85] and the default parameters except for -score 80. We estimated the pairwise dN/dS for each core gene using the approach described previously and estimated the median dN/dS for our genus-representative genomes. A linear regression between the dN/dS values resulting from core genes and the 120 marker genes set for the 40 genera showed similar results (S2 Fig), therefore we used the latter for further statistical analyses. In addition, we predicted ribosomal RNA genes in our representative genomes as an approximation to ecological strategy using Barrnap with the default parameters (barrnap 0.9: rapid ribosomal RNA prediction; https://github.com/tseemann/barrnap). Genome size, 16S rRNA copies, and dN/dS values for the GTDB representative genomes dataset are reported in S2 Data.

### Statistical analyses

Due to the tendency of related species to resemble each other because of their shared phylogenetic ancestry, we assessed the suitability of a phylogeny-based method for our regression analyses by first estimating Blomberg’s K on genome size data [35] using the phylosignal function on R [86]. This parameter represents the phylogenetic signal in a continuous trait, and goes from 0 (no phylogenetic signal) to ∞ (phylogenetic signal) with the null hypothesis (K = 1) meaning that the trait analyzed evolves under Brownian Motion [40,87]. In addition, we also tested the fit of different trait evolution models, including Brownian Motion [40], Ornstein-Uhlenbeck [41], Early-Burst [42], a diffusion model, Pagel’s model [43], a drift model, and a white-noise model (non-phylogenetic). We tested the predictability of genome size from dN/dS and 16S rRNA copies as predictor variables using the “glm” function available on R. Since we detected phylogenetic signal in genome size data, we additionally accounted for potential phylogenetic nonindependence in the residuals using the PGLS method with the function pgls on the R package Caper [88] and the Pagel’s model [43], as well as the function gls available on the package ape [89]. We additionally tested the effect of sample size on the calculation of Blomberg’s K and Pagel’s lambda by estimating these parameters within each phylum (Figs 1A and S3). The trait data and the phylogeny used in these analyses can be found in S2 Data and S1 Phylogeny, respectively. In addition to testing phylogenetic signal in a broad-scale phylogeny (S2 Data, Fig 2), we built a phylogenetic tree with multiple representative genomes for each genus and the IQ-TREE workflow used for the rest of our analyses. The genome size data and the phylogenetic tree used for this analysis can be found in S1 Data and S2 Phylogeny, respectively.

## Supporting information

S1 Data Genomes used to calculate pairwise dN/dS within each genus cluster. (TSV)

S2 Data dN/dS, genome size, and *rrn* operon copies for each genus representative. (TSV)

S1 Fig Relationship between mean genome size and the difference in Mbp between the largest and shortest genome for each genus cluster. Abbreviations: TDS = Thermotogota, Deinococcota, and Synergistota. Raw data for genome size can be found in S3 Data.
(TIF)

S2 Fig Regression between the dN/dS estimates resulting from 120 and 122 markers genes for bacteria and archaea respectively, and the core genes of 40 randomly selected genera from the GTDB genomes dataset. (TIF)

S3 Fig Pagel’s lambda and Blomberg’s K estimation within GTDB genomes dataset phyla. (TIF)

S1 Table Summary of model fitting for genome size data using a fine scale phylogeny (multiple representatives per genus). We highlighted the model that showed the highest likelihood and the lowest AIC.
(DOCX)

S1 Phylogeny Broad scale phylogeny (one representative per genus) used to perform the phylogenetic signal estimation on genome size data and the phylogenetic generalized least square tests. (TXT)

S2 Phylogeny Fine scale phylogeny (multiple representatives per genus) used to perform the phylogenetic signal estimation on genome size data. (TXT)

## References

[1] Mira A, Ochman H, Moran NA. Deletional bias and the evolution of bacterial genomes. Trends Genet. 2001;17: 589–596. doi: 10.1016/s0168-9525(01)02447-7 11585665

[2] Lynch M. Streamlining and simplification of microbial genome architecture. Annu Rev Microbiol. 2006;60: 327–349. doi: 10.1146/annurev.micro.60.080805.142300 16824010

[3] Koonin EV. Evolution of genome architecture. Int J Biochem Cell Biol. 2009;41: 298–306. doi: 10.1016/j.biocel.2008.09.015 18929678 PMC3272702

[4] Lawrence JG, Hendrix RW, Casjens S. Where are the pseudogenes in bacterial genomes? Trends Microbiol. 2001;9: 535–540. doi: 10.1016/s0966-842x(01)02198-9 11825713

[5] Wolf YI, Aravind L, Grishin NV, Koonin EV. Evolution of aminoacyl-tRNA synthetases—analysis of unique domain architectures and phylogenetic trees reveals a complex history of horizontal gene transfer events. Genome Res. 1999;9: 689–710. 10447505

[6] Bobay L-M, Ochman H. The Evolution of Bacterial Genome Architecture. Front Genet. 2017;8: 72. doi: 10.3389/fgene.2017.00072 28611826 PMC5447742

[7] Andreani NA, Hesse E, Vos M. Prokaryote genome fluidity is dependent on effective population size. ISME J. 2017;11: 1719–1721. doi: 10.1038/ismej.2017.36 28362722 PMC5520154

[8] Sela I, Wolf YI, Koonin EV. Theory of prokaryotic genome evolution. Proc Natl Acad Sci U S A. 2016;113: 11399–11407. doi: 10.1073/pnas.1614083113 27702904 PMC5068321

[9] van Ham RCHJ, Kamerbeek J, Palacios C, Rausell C, Abascal F, Bastolla U, et al. Reductive genome evolution in Buchnera aphidicola. Proc Natl Acad Sci U S A. 2003;100: 581–586. doi: 10.1073/pnas.0235981100 12522265 PMC141039

[10] Moran NA, Mira A. The process of genome shrinkage in the obligate symbiont Buchnera aphidicola. Genome Biol. 2001;2: RESEARCH0054. doi: 10.1186/gb-2001-2-12-research0054 11790257 PMC64839

[11] Chong RA, Park H, Moran NA. Genome Evolution of the Obligate Endosymbiont Buchnera aphidicola. Mol Biol Evol. 2019;36: 1481–1489. doi: 10.1093/molbev/msz082 30989224

[12] Batut B, Knibbe C, Marais G, Daubin V. Reductive genome evolution at both ends of the bacterial population size spectrum. Nat Rev Microbiol. 2014;12: 841–850. doi: 10.1038/nrmicro3331 25220308

[13] Woolfit M, Bromham L. Increased rates of sequence evolution in endosymbiotic bacteria and fungi with small effective population sizes. Mol Biol Evol. 2003;20: 1545–1555. doi: 10.1093/molbev/msg167 12832648

[14] Biller SJ, Berube PM, Lindell D, Chisholm SW. Prochlorococcus: the structure and function of collective diversity. Nat Rev Microbiol. 2015;13: 13–27. doi: 10.1038/nrmicro3378 25435307

[15] Kashtan N, Roggensack SE, Rodrigue S, Thompson JW, Biller SJ, Coe A, et al. Single-cell genomics reveals hundreds of coexisting subpopulations in wild Prochlorococcus. Science. 2014;344: 416–420. doi: 10.1126/science.1248575 24763590

[16] Grote J, Thrash JC, Huggett MJ, Landry ZC, Carini P, Giovannoni SJ, et al. Streamlining and core genome conservation among highly divergent members of the SAR11 clade. MBio. 2012;3. doi: 10.1128/mBio.00252-12 22991429 PMC3448164

[17] Giovannoni SJ, Cameron Thrash J, Temperton B. Implications of streamlining theory for microbial ecology. ISME J. 2014;8: 1553–1565. doi: 10.1038/ismej.2014.60 24739623 PMC4817614

[18] Simonsen AK. Environmental stress leads to genome streamlining in a widely distributed species of soil bacteria. The ISME Journal. 2021. doi: 10.1038/s41396-021-01082-x 34408268 PMC8776746

[19] Rodríguez-Gijón A, Nuy JK, Mehrshad M, Buck M, Schulz F, Woyke T, Garcia SL. A Genomic Perspective Across Earth’s Microbiomes Reveals That Genome Size in Archaea and Bacteria Is Linked to Ecosystem Type and Trophic Strategy. Frontiers in Microbiol. 2021;12: 761869–761869. doi: 10.3389/fmicb.2021.761869 35069467 PMC8767057

[20] Nielsen DA, Fierer N, Geoghegan JL, Gillings MR, Gumerov V, Madin JS, et al. Aerobic bacteria and archaea tend to have larger and more versatile genomes. Oikos. 2021. pp. 501–511. doi: 10.1111/oik.07912

[21] Bourguignon T, Kinjo Y, Villa-Martín P, Coleman NV, Tang Q, Arab DA, et al. Increased Mutation Rate Is Linked to Genome Reduction in Prokaryotes. Curr Biol. 2020;30: 3848–3855.e4. doi: 10.1016/j.cub.2020.07.034 32763167

[22] Marais GAB, Calteau A, Tenaillon O. Mutation rate and genome reduction in endosymbiotic and free-living bacteria. Genetica. 2008;134: 205–210. doi: 10.1007/s10709-007-9226-6 18046510

[23] Marais GAB, Batut B, Daubin V. Genome Evolution: Mutation Is the Main Driver of Genome Size in Prokaryotes. Curr Biol. 2020;30: R1083–R1085. doi: 10.1016/j.cub.2020.07.093 33022240

[24] Osburne MS, Holmbeck BM, Coe A, Chisholm SW. The spontaneous mutation frequencies of Prochlorococcus strains are commensurate with those of other bacteria. Environ Microbiol Rep. 2011;3: 744–749. doi: 10.1111/j.1758-2229.2011.00293.x 23761365

[25] Chen Z, Wang X, Song Y, Zeng Q, Zhang Y, Luo H. Prochlorococcus have low global mutation rate and small effective population size. Nature Ecology & Evolution. 2021. doi: 10.1038/s41559-021-01591-0 34949817

[26] Felsenstein J. Phylogenies and the Comparative Method. The American Naturalist. 1985. pp. 1–15. doi: 10.1086/284325 31094602

[27] Garland T, Midford PE, Ives AR. An Introduction to Phylogenetically Based Statistical Methods, with a New Method for Confidence Intervals on Ancestral Values. American Zoologist. 1999. pp. 374–388. doi: 10.1093/icb/39.2.374

[28] Chaumeil P-A, Mussig AJ, Hugenholtz P, Parks DH. GTDB-Tk: a toolkit to classify genomes with the Genome Taxonomy Database. Bioinformatics. 2019. doi: 10.1093/bioinformatics/btz848 31730192 PMC7703759

[29] Parks DH, Chuvochina M, Chaumeil P-A, Rinke C, Mussig AJ, Hugenholtz P. A complete domain-to-species taxonomy for Bacteria and Archaea. Nat Biotechnol. 2020;38: 1079–1086. doi: 10.1038/s41587-020-0501-8 32341564

[30] Martinez-Gutierrez CA, Aylward FO. Phylogenetic Signal, Congruence, and Uncertainty across Bacteria and Archaea. Mol Biol Evol. 2021. doi: 10.1093/molbev/msab254 34436605 PMC8662615

[31] Dobrindt U, Hacker J. Whole Genome Plasticity in Pathogenic Bacteria. 2001. 10.1016/s1369-5274(00)00250-2 11587932

[32] Lawrence JG, Hendrickson H. Genome evolution in bacteria: order beneath chaos. Curr Opin Microbiol. 2005;8: 572–578. doi: 10.1016/j.mib.2005.08.005 16122972

[33] Lan R, Reeves PR. Intraspecies variation in bacterial genomes: the need for a species genome concept. Trends Microbiol. 2000;8: 396–401. doi: 10.1016/s0966-842x(00)01791-1 10989306

[34] Casjens S. The diverse and dynamic structure of bacterial genomes. Annu Rev Genet. 1998;32: 339–377. doi: 10.1146/annurev.genet.32.1.339 9928484

[35] Blomberg SP, Garland T Jr, Ives AR. Testing for phylogenetic signal in comparative data: behavioral traits are more labile. Evolution. 2003;57: 717–745. doi: 10.1111/j.0014-3820.2003.tb00285.x 12778543

[36] Münkemüller T, Lavergne S, Bzeznik B, Dray S, Jombart T, Schiffers K, et al. How to measure and test phylogenetic signal. Methods in Ecology and Evolution. 2012. pp. 743–756. doi: 10.1111/j.2041-210x.2012.00196.x

[37] Revell LJ, Harmon LJ, Collar DC. Phylogenetic signal, evolutionary process, and rate. Syst Biol. 2008;57: 591–601. doi: 10.1080/10635150802302427 18709597

[38] Freckleton Freckleton, Harvey Pagel. Phylogenetic Analysis and Comparative Data: A Test and Review of Evidence. The American Naturalist. 2002. p. 712. doi: 10.1086/343873 18707460

[39] Revell LJ. Phylogenetic signal and linear regression on species data. Methods in Ecology and Evolution. 2010. pp. 319–329. doi: 10.1111/j.2041-210x.2010.00044.x

[40] Felsenstein J. Maximum-likelihood estimation of evolutionary trees from continuous characters. Am J Hum Genet. 1973;25: 471–492. 4741844 PMC1762641

[41] Butler MA, King AA. Phylogenetic Comparative Analysis: A Modeling Approach for Adaptive Evolution. Am Nat. 2004;164: 683–695. doi: 10.1086/426002 29641928

[42] Harmon LJ, Losos JB, Jonathan Davies T, Gillespie RG, Gittleman JL, Bryan Jennings W, et al. Early bursts of body size and shape evolution are rare in comparative data. Evolution. 2010;64: 2385–2396. doi: 10.1111/j.1558-5646.2010.01025.x 20455932

[43] Pagel M. Inferring the historical patterns of biological evolution. Nature. 1999. pp. 877–884. doi: 10.1038/44766 10553904

[44] Molina-Venegas R, Rodríguez MÁ. Revisiting phylogenetic signal; strong or negligible impacts of polytomies and branch length information? BMC Evol Biol. 2017;17: 53. doi: 10.1186/s12862-017-0898-y 28201989 PMC5312541

[45] Hernández CE, Rodríguez-Serrano E, Avaria-Llautureo J, Inostroza-Michael O, Morales-Pallero B, Boric-Bargetto D, et al. Using phylogenetic information and the comparative method to evaluate hypotheses in macroecology. Methods in Ecology and Evolution. 2013. pp. 401–415. doi: 10.1111/2041-210x.12033

[46] Kamilar JM, Cooper N. Phylogenetic signal in primate behaviour, ecology and life history. Philosophical Transactions of the Royal Society B: Biological Sciences. 2013. doi: 10.1098/rstb.2012.0341 PMC3638444 23569289

[47] Kuo C-H, Moran NA, Ochman H. The consequences of genetic drift for bacterial genome complexity. Genome Res. 2009;19: 1450–1454. doi: 10.1101/gr.091785.109 19502381 PMC2720180

[48] Martinez-Gutierrez CA, Aylward FO. Strong Purifying Selection Is Associated with Genome Streamlining in Epipelagic Marinimicrobia. Genome Biol Evol. 2019;11: 2887–2894. doi: 10.1093/gbe/evz201 31539038 PMC6798728

[49] Whitney KD, Garland T Jr. Did genetic drift drive increases in genome complexity? PLoS Genet. 2010;6. doi: 10.1371/journal.pgen.1001080 20865118 PMC2928810

[50] Whitney KD, Boussau B, Baack EJ, Garland T Jr. Drift and genome complexity revisited. PLoS Genet. 2011;7: e1002092. doi: 10.1371/journal.pgen.1002092 21695239 PMC3111538

[51] Revell LJ, Collar DC. PHYLOGENETIC ANALYSIS OF THE EVOLUTIONARY CORRELATION USING LIKELIHOOD. Evolution. 2009. pp. 1090–1100. doi: 10.1111/j.1558-5646.2009.00616.x 19154380

[52] Rocha EPC, Smith JM, Hurst LD, Holden MTG, Cooper JE, Smith NH, et al. Comparisons of dN/dS are time dependent for closely related bacterial genomes. J Theor Biol. 2006;239: 226–235. doi: 10.1016/j.jtbi.2005.08.037 16239014

[53] Luo H, Huang Y, Stepanauskas R, Tang J. Excess of non-conservative amino acid changes in marine bacterioplankton lineages with reduced genomes. Nat Microbiol. 2017;2: 17091. doi: 10.1038/nmicrobiol.2017.91 28604700

[54] Klappenbach JA, Dunbar JM, Schmidt TM. rRNA operon copy number reflects ecological strategies of bacteria. Appl Environ Microbiol. 2000;66: 1328–1333. doi: 10.1128/AEM.66.4.1328-1333.2000 10742207 PMC91988

[55] Niederdorfer R, Besemer K, Battin TJ, Peter H. Ecological strategies and metabolic trade-offs of complex environmental biofilms. NPJ Biofilms Microbiomes. 2017;3: 21. doi: 10.1038/s41522-017-0029-y 28955480 PMC5612939

[56] Condon C, Liveris D, Squires C, Schwartz I, Squires CL. rRNA operon multiplicity in Escherichia coli and the physiological implications of rrn inactivation. J Bacteriol. 1995;177: 4152–4156. doi: 10.1128/jb.177.14.4152-4156.1995 7608093 PMC177152

[57] Chuckran PF, Hungate BA, Schwartz E, Dijkstra P. Variation in genomic traits of microbial communities among ecosystems. FEMS Microbes. 2022. doi: 10.1093/femsmc/xtab020 PMC10117780 37334231

[58] Guieysse B, Wuertz S. Metabolically versatile large-genome prokaryotes. Curr Opin Biotechnol. 2012;23: 467–473. doi: 10.1016/j.copbio.2011.12.022 22226959

[59] Lynch M, Conery JS. The Origins of Genome Complexity. Science. 2003. pp. 1401–1404. doi: 10.1126/science.1089370 14631042

[60] Konstantinidis KT, Tiedje JM. Trends between gene content and genome size in prokaryotic species with larger genomes. Proc Natl Acad Sci U S A. 2004;101: 3160–3165. doi: 10.1073/pnas.0308653100 14973198 PMC365760

[61] Graham CH, Storch D, Machac A. Phylogenetic scale in ecology and evolution. doi: 10.1101/063560

[62] Tamas I, Klasson L, Canbäck B, Näslund AK, Eriksson A-S, Wernegreen JJ, et al. 50 million years of genomic stasis in endosymbiotic bacteria. Science. 2002;296: 2376–2379. doi: 10.1126/science.1071278 12089438

[63] McCutcheon JP, Moran NA. Extreme genome reduction in symbiotic bacteria. Nat Rev Microbiol. 2011;10: 13–26. doi: 10.1038/nrmicro2670 22064560

[64] López-Pérez M, Haro-Moreno JM, Coutinho FH, Martinez-Garcia M, Rodriguez-Valera F. The Evolutionary Success of the Marine Bacterium SAR11 Analyzed through a Metagenomic Perspective. mSystems. 2020;5. doi: 10.1128/mSystems.00605-20 33024052 PMC7542561

[65] Giovannoni SJ. SAR11 Bacteria: The Most Abundant Plankton in the Oceans. Ann Rev Mar Sci. 2017;9: 231–255. doi: 10.1146/annurev-marine-010814-015934 27687974

[66] Partensky F, Garczarek L. Prochlorococcus: advantages and limits of minimalism. Ann Rev Mar Sci. 2010;2: 305–331. doi: 10.1146/annurev-marine-120308-081034 21141667

[67] Cottrell MT, Kirchman DL. Transcriptional Control in Marine Copiotrophic and Oligotrophic Bacteria with Streamlined Genomes. Appl Environ Microbiol. 2016;82: 6010–6018. doi: 10.1128/AEM.01299-16 27474718 PMC5038029

[68] Carini P, Steindler L, Beszteri S, Giovannoni SJ. Nutrient requirements for growth of the extreme oligotroph “Candidatus Pelagibacter ubique” HTCC1062 on a defined medium. The ISME Journal. 2013. pp. 592–602. doi: 10.1038/ismej.2012.122 23096402 PMC3578571

[69] Fierer N. Embracing the unknown: disentangling the complexities of the soil microbiome. Nat Rev Microbiol. 2017;15: 579–590. doi: 10.1038/nrmicro.2017.87 28824177

[70] Takeuchi N, Cordero OX, Koonin EV, Kaneko K. Gene-specific selective sweeps in bacteria and archaea caused by negative frequency-dependent selection. BMC Biol. 2015;13: 20. doi: 10.1186/s12915-015-0131-7 25928466 PMC4410459

[71] Goldman B, Bhat S, Shimkets LJ. Genome evolution and the emergence of fruiting body development in Myxococcus xanthus. PLoS One. 2007;2: e1329. doi: 10.1371/journal.pone.0001329 18159227 PMC2129111

[72] Sunagawa S, Mende DR, Zeller G, Izquierdo-Carrasco F, Berger SA, Kultima JR, et al. Metagenomic species profiling using universal phylogenetic marker genes. Nat Methods. 2013;10: 1196–1199. doi: 10.1038/nmeth.2693 24141494

[73] Capella-Gutiérrez S, Silla-Martínez JM, Gabaldón T. trimAl: a tool for automated alignment trimming in large-scale phylogenetic analyses. Bioinformatics. 2009;25: 1972–1973. doi: 10.1093/bioinformatics/btp348 19505945 PMC2712344

[74] Nguyen L-T, Schmidt HA, von Haeseler A, Minh BQ. IQ-TREE: a fast and effective stochastic algorithm for estimating maximum-likelihood phylogenies. Mol Biol Evol. 2015;32: 268–274. doi: 10.1093/molbev/msu300 25371430 PMC4271533

[75] Minh BQ, Nguyen MAT, von Haeseler A. Ultrafast approximation for phylogenetic bootstrap. Mol Biol Evol. 2013;30: 1188–1195. doi: 10.1093/molbev/mst024 23418397 PMC3670741

[76] Le SQ, Gascuel O. An improved general amino acid replacement matrix. Mol Biol Evol. 2008;25: 1307–1320. doi: 10.1093/molbev/msn067 18367465

[77] Soubrier J, Steel M, Lee MSY, Der Sarkissian C, Guindon S, Ho SYW, et al. The influence of rate heterogeneity among sites on the time dependence of molecular rates. Mol Biol Evol. 2012;29: 3345–3358. doi: 10.1093/molbev/mss140 22617951

[78] Letunic I, Bork P. Interactive Tree Of Life (iTOL) v4: recent updates and new developments. Nucleic Acids Res. 2019;47: W256–W259. doi: 10.1093/nar/gkz239 30931475 PMC6602468

[79] Parks DH, Imelfort M, Skennerton CT, Hugenholtz P, Tyson GW. CheckM: assessing the quality of microbial genomes recovered from isolates, single cells, and metagenomes. Genome Res. 2015;25: 1043–1055. doi: 10.1101/gr.186072.114 25977477 PMC4484387

[80] Eddy SR. Accelerated Profile HMM Searches. PLoS Comput Biol. 2011;7: e1002195. doi: 10.1371/journal.pcbi.1002195 22039361 PMC3197634

[81] Sievers F, Higgins DG. Clustal Omega for making accurate alignments of many protein sequences. Protein Science. 2018. pp. 135–145. doi: 10.1002/pro.3290 28884485 PMC5734385

[82] Suyama M, Torrents D, Bork P. PAL2NAL: robust conversion of protein sequence alignments into the corresponding codon alignments. Nucleic Acids Research. 2006. pp. W609–W612. doi: 10.1093/nar/gkl315 16845082 PMC1538804

[83] Yang Z. PAML 4: phylogenetic analysis by maximum likelihood. Mol Biol Evol. 2007;24: 1586–1591. doi: 10.1093/molbev/msm088 17483113

[84] Harris CD, Torrance EL, Raymann K, Bobay L-M. CoreCruncher: Fast and Robust Construction of Core Genomes in Large Prokaryotic Data Sets. Mol Biol Evol. 2021;38: 727–734. doi: 10.1093/molbev/msaa224 32886787 PMC7826169

[85] Edgar RC. Search and clustering orders of magnitude faster than BLAST. Bioinformatics. 2010. pp. 2460–2461. doi: 10.1093/bioinformatics/btq461 20709691

[86] Kembel SW, Cowan PD, Helmus MR, Cornwell WK, Morlon H, Ackerly DD, et al. Picante: R tools for integrating phylogenies and ecology. Bioinformatics. 2010;26: 1463–1464. doi: 10.1093/bioinformatics/btq166 20395285

[87] O’Meara BC, Ané C, Sanderson MJ, Wainwright PC. Testing for different rates of continuous trait evolution using likelihood. Evolution. 2006;60: 922–933. 16817533

[88] Website. Available: Orme D, Freckleton R, Thomas G, Petzold T, Fritz S, Isaac N, Pears W. 2012. Caper: Comparative Analyses of Phylogenetics and Evolution in R. Version 0.5. [WWW document] URL http://cran.r-project.org/web/packages/caper/caper.pdf [accessed 25 April 2013].

[89] Paradis E, Schliep K. ape 5.0: an environment for modern phylogenetics and evolutionary analyses in R. Bioinformatics. 2019. pp. 526–528. doi: 10.1093/bioinformatics/bty633 30016406

