# Supplementary figures and images for: Genome size distributions in bacteria and archaea are strongly linked to evolutionary history at broad phylogenetic scales
Source: PLoS Genet. 2022 May 23;18(5):e1010220. doi: 10.1371/journal.pgen.1010220 (PMC9166353; doi:10.1371/journal.pgen.1010220)

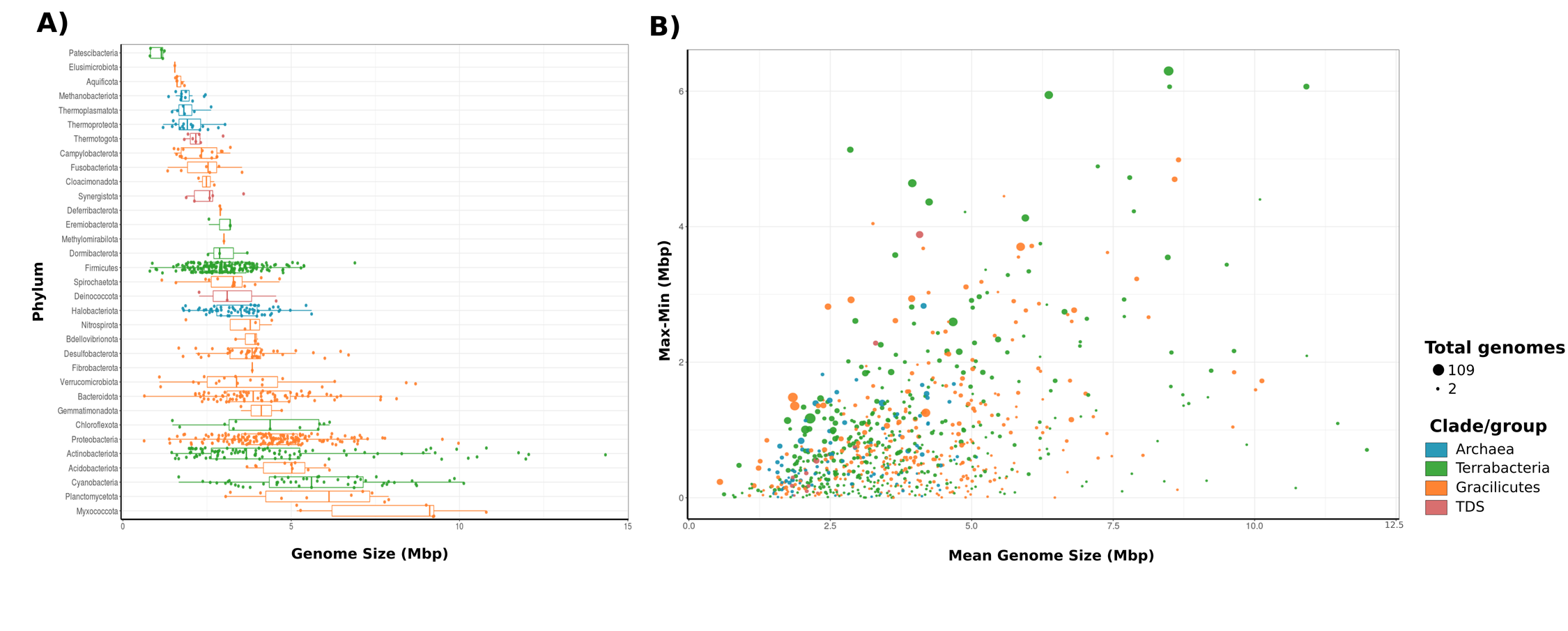

Supplement: S1 Fig — Abbreviations: TDS = Thermotogota, Deinococcota, and Synergistota. Raw data for genome size can be found in S3 Data. (TIF) [file pgen.1010220.s003.tif]

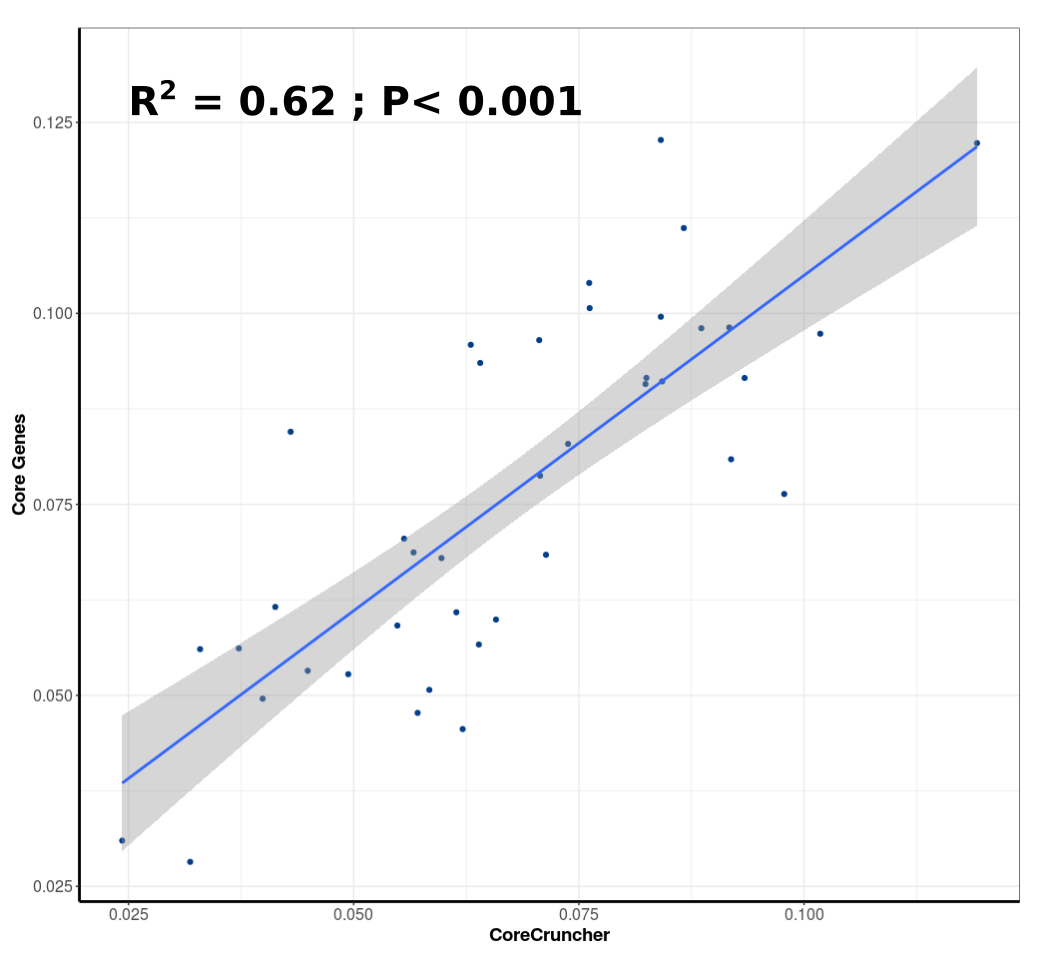

Supplement: S2 Fig — (TIF) [file pgen.1010220.s004.tif]

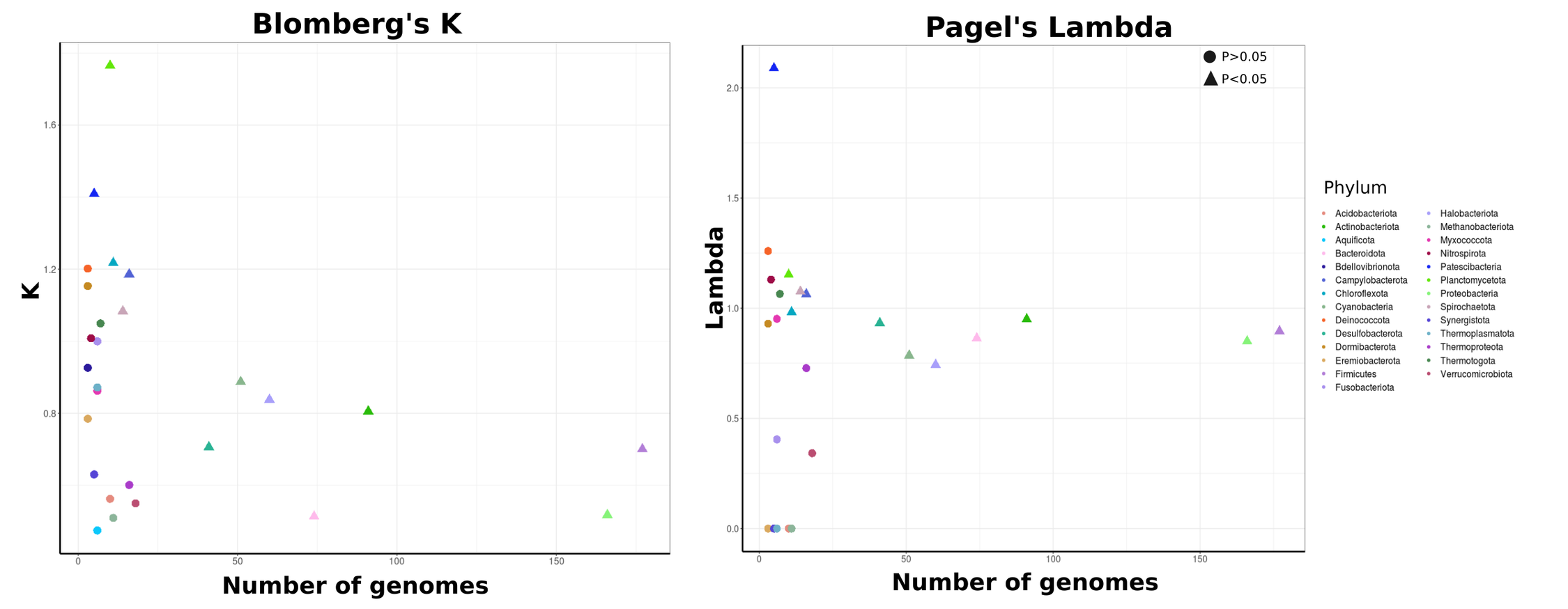

Supplement: S3 Fig — (TIF) [file pgen.1010220.s005.tif]
