# Supplementary material for: Genome size distributions in bacteria and archaea are strongly linked to evolutionary history at broad phylogenetic scales
Source: PLoS Genet. 2022 May 23;18(5):e1010220. doi: 10.1371/journal.pgen.1010220 (PMC9166353; doi:10.1371/journal.pgen.1010220)
Supplement: S1 Table — We highlighted the model that showed the highest likelihood and the lowest AIC. (DOCX) [file pgen.1010220.s006.docx]

| Model | Loglik | Parameters | AIC |
| --- | --- | --- | --- |
| Brownian motion | -5320.4 | Sigma = 12.8  Root state = 2.7 | 10644.8 |
| Ornstein-Uhlenbeck | -5152.5 | alpha = 2.7  Sigma = 14.9  Root state = 3.1 | 10311 |
| Early-Burst | -5320.4 | a = 0  Sigma = 12.8  Root state = 2.7 | 10646.8 |
| Pagel’s model* | -4913.2 | **Lambda = 0.99**  Sigma = 6.7  Root state = 2.7 | 9832.4 |
| Trend diffusion | -5265.1 | Slope = 100  Sigma = 0.1  Root state = 2.8 | 10536.2 |
| Drift | -5320.4 | Drift = 100  Sigma = 12.8  Root state = -97.3 | 10646.8 |
| White-noise | -9284.5 | Sigma = 4.1  Root state = 3.9 | 18573 |

*Significantly higher likelihood when compared with the rest of the models tested according to the chisq test (P<0.001)
